# Supplementary material for: Circular RNA biogenesis can proceed through an exon-containing lariat precursor
Source: eLife. 2015 Jun 9;4:e07540. doi: 10.7554/eLife.07540 (PMC4479058; doi:10.7554/eLife.07540)
Supplement: Supplementary file 1. — List of primers used for cloning in this study. DOI: http://dx.doi.org/10.7554/eLife.07540.023 [file elife07540s001.pdf]

Supplementary file 1

**QuikChange primers**	
**mrps16_5ss1QC_fwd**	CGTAGTTGCGAATGCATGAGGATGAAAACATACC
**mrps16_5ss1QC_rev**	GGTATGTTTTCATCCTCATGCATTCGCAACTACG
**mrps16_3ss1QC_fwd**	CGACTTTGTTAACTCAATATGAAAGCACCTCAAGCC
**mrps16_3ss1QC_rev**	GGCTTGAGGTGCTTTCATATTGAGTTAACAAAGTCG
**mrps16_bp1QC_fwd**	GGTTCGACTTTG TCAATATAGGAAAGCACCTC
**mrps16_bp1QC_rev**	GAGGTGCTTTCCTATATTGACAAAGTCGAACC
**mrps16_5ss2QC_fwd**	CCGCTCTTTAGCTGAAAAGCCAAGCTCTC
**mrps16_5ss2QC_rev**	GAGAGCTTGGCTTTTCAGCTAAAGAGCGG
**mrps16_3ss2QC_fwd**	CTTTTTTCACTTTTTTCCAGTTGCTTCCTAAAAAACCCG
**mrps16_3ss2QC_rev**	CGGGTTTTTTAGGAAGCAACTGGAAAAAAGTGAAAAAAG
**mrps16_bp2QC_fwd**	CTATCAATTCTTGTTACTTTTTTCACTTTTAGTTCCAGTTGC
**mrps16_bp2QC_rev**	GCAACTGGAACTAAAAGTGAAAAAAGTAACAAGAATTGATAG
**pub1_bp3QC_fwd**	GATTAAAAACAATGTTTAATTCTTTAGTAATGTTACAGATAATAG CACC
**pub1_bp3QC_rev**	GGTGCTATTATCTGTAACATTACTAAAGAATTAAACATTGTTTTTA ATC
**mrps16_Δ90_Exon2QC_fwd**	CCTATCGAAACCATCGGCACTTTTGGTGTAGGAGCACAACCATCC GACAC
**mrps16_Δ90_Exon2QC_rev**	GTGTCGGATGGTTGTGCTCCTACACCAAAAGTGCCGATGGTTTCG ATAGG
***mrps16* exon deletion library primers**	
**mrps16_del_col_fwd_1**	AGCTGAAAAGGTATGCCCA
**mrps16_del_col_fwd_2**	CCGTCCGCTCTTTAGCTG
**mrps16_del_col_fwd_3**	CAACCATCCGACACCGT
**mrps16_del_col_fwd_4**	TTGGATAAGTGTAGGAGCACAA
**mrps16_del_col_fwd_5**	ACGTTGAACGTTTTAAATATTGGATAA
**mrps16_del_col_fwd_6**	CAGCTCAACGTTGAACGTTT
**mrps16_del_col_fwd_7**	TCCTCGTATAAAAGATATCCAGCTC
**mrps16_del_col_fwd_8**	CAGGACTCCATTCCTCGTATAA
**mrps16_del_col_fwd_9**	CTAAGAAGATTGATTCTCAGGACTC
**mrps16_del_col_fwd_10**	TTGATCCCATCCCTAAGAAGATT
**mrps16_del_col_fwd_11**	ATCGGCACTTTTGATCCCA
**mrps16_del_col_fwd_12**	CAAACCTATCGAAACCATCGG
**mrps16_del_col_rev_1**	AGGTGCTTTCCTATATTGAGTTAAC
**mrps16_del_col_rev_2**	TGGCTTGAGGTGCTTTCC
**mrps16_del_col_rev_3**	GATGGTTTCGATAGGTTTGGC
**mrps16_del_col_rev_4**	GATCAAAAGTGCCGATGGTT
**mrps16_del_col_rev_5**	AATCTTCTTAGGGATGGGATCAA
**mrps16_del_col_rev_6**	GGAGTCCTGAGAATCAATCTTCTT
**mrps16_del_col_rev_7**	ACGAGGAATGGAGTCCTGA
**mrps16_del_col_rev_8**	GTTGAGCTGGATATCTTTTATACGAG
**mrps16_del_col_rev_9**	CGTTCAACGTTGAGCTGGAT
**mrps16_del_col_rev_10**	CTACACTTATCCAATATTTAAAACGTTCA
**mrps16_del_col_rev_11**	TGTGCTCCTACACTTATCCAAT
**mrps16_del_col_rev_12**	GTCGGATGGTTGTGCTCC
